# Tamoxifen resistance-related ceRNA network for breast cancer

**DOI:** 10.3389/fcell.2022.1023079

**Published:** 2022-11-25

**Authors:** Zipeng Qiao, Yu Xing, Qingquan Zhang, Yongjun Tang, Ruifa Feng, Weiyi Pang

**Affiliations:** ^1^ Guangxi Key Laboratory of Environmental Exposomics and Entire Lifecycle Heath, Guilin Medical University, Guilin, Guangxi, China; ^2^ School of Public Health, Guilin Medical University, Guilin, Guangxi, China; ^3^ The Second Affiliated Hospital of Guilin Medical University, Guilin, Guangxi, China; ^4^ School of Humanities and Management, Guilin Medical University, Guilin, Guangxi, China

**Keywords:** bioinformatics, breast cancer, ceRNA, TMX resistant, lncRNA, prognostic

## Abstract

**Background:** Tamoxifen (TMX) is one of the most widely used drugs to treat breast cancer (BC). However, acquired drug resistance is still a major obstacle to its application, rendering it crucial to explore the mechanisms of TMX resistance in BC. This aims of this study were to identify the mechanisms of TMX resistance and construct ceRNA regulatory networks in breast cancer.

**Methods:** GEO2R was used to screen for differentially expressed mRNAs (DEmRNAs) leading to drug resistance in BC cells. MiRTarbase and miRNet were used to predict miRNAs and lncRNAs upstream, and the competing endogenous RNA (ceRNA) regulatory network of BC cell resistance was constructed by starBase. We used the Kaplan–Meier plotter and Gene Expression Profiling Interactive Analysis (GEPIA) to analyze the expression and prognostic differences of genes in the ceRNA network with core axis, and qRT-PCR was used to further verify the above conclusions.

**Results:** We found that 21 DEmRNAs were upregulated and 43 DEmRNA downregulated in drug-resistant BC cells. DEmRNAs were noticeably enriched in pathways relevant to cancer. We then constructed a protein-protein interaction (PPI) network based on the STRING database and defined 10 top-ranked hub genes among the upregulated and downregulated DEmRNAs. The 20 DEmRNAs were predicted to obtain 113 upstream miRNAs and 501 lncRNAs. Among them, 7 mRNAs, 22 lncRNAs, and 11 miRNAs were used to structure the ceRNA regulatory network of drug resistance in BC cells. 4 mRNAs, 4 lncRNAs, and 3 miRNAs were detected by GEPIA and the Kaplan–Meier plotter to be significantly associated with BC expression and prognosis. The differential expression of the genes in BC cells was confirmed by qRT-PCR.

**Conclusion:** The ceRNA regulatory network of TMX-resistant BC was successfully constructed and confirmed. This will provide an important resource for finding therapeutic targets for TMX resistance, where the discovery of candidate conventional mechanisms can aid clinical decision-making. In addition, this resource will help discover the mechanisms behind this type of resistance.

## Introduction

The high recurrence rate of breast cancer (BC) is a serious threat to women’s health, causing deterioration of their quality of life. BCs are classified as human epidermal growth factor receptor 2 (Her-2), luminal A, luminal B, and triple negative BC, according to the status of the progesterone receptor (PR), estrogen receptor (ER) and Her-2 receptor (Yeo and Guan, 2017). Approximately 75% of diagnosed BC cases are ER-positive (Asif et al., 2016). The predominant treatment for ER-positive BC is currently an adjunctive endocrine approach, such as with TMX and letrozole, which has a great effect on improving BC survival and reducing the risk of recurrence (Lambert et al., 2018). However, due to heterogeneity, breast tumors often exhibit high recurrence rates and drug resistance, and there is approximately a 50% failure rate for TMX in the clinical treatment of patients with advanced ER-positive BC (Natarajan et al., 2012; Jia et al., 2015). There is an urgent need to develop personalized BC treatment and prevention strategies since the mechanism of action of acquired drug-resistant responses is unknown. Therefore, screening for biomarkers leading to the development of drug resistance in ER-positive BC plays a key role in early diagnosis, BC recurrence and improved prognosis.

An increasing number of studies confirm that competitive endogenous RNAs (ceRNAs) play a critical role in the development of cells since Salmena et al. first proposed the ceRNA hypothesis (Salmena et al., 2011; Wang et al., 2021a; Behera et al., 2021). With further exploration and refinement, the role of ceRNAs has attracted more attention in BC drug resistance formation (Dong et al., 2018; Yao et al., 2019; Yan et al., 2020). Jiang et al. showed that lncRNA-linc01561 increased MMP11 expression and promoted BC cell growth by sponging miR-145-5p, while inhibition of linc01561 expression also decreased MMP11 expression thereby inhibiting BC cell growth (Jiang et al., 2018). Similarly, Tang et al. showed that miR-145-5p could also target binding to SOX2 to interfere with the growth of BC cells (Tang et al., 2019). Du et al. found that lncRNA DLX6-AS1 promotes the epithelial-mesenchymal transition process in triple-negative BC by regulating the mir-199b-5p/PXN axis and enhances cisplatin resistance of the cells (Du et al., 2020). Li et al. showed that LINC00680 inhibits the growth of BC cells by affecting the miR-320b/CDKL5 axis of cell proliferation and invasion, promoting doxorubicin resistance in BC cells (Li et al., 2022).

It is clear ceRNAs are playing an increasingly important role in drug resistance in BC. In our study, we screened the differentially expressed genes leading to TMX-resistant BC by GEO2R and constructed a ceRNA network associated with TMX-resistant BC. We continued to screen the TMX-resistant ceRNA network for differentially expressed genes associated with BC prognosis by GEPIA and the Kaplan–Meier plotter. Finally, we obtained an axis associated with both BC drug resistance and prognosis, which was validated by qRT-PCR.

## Materials and methods

### Gene expression data collection and screening

To determine the differential gene expression profile of TMX-resistant BC, we screened the dataset of TMX-resistant BC samples from the Gene Expression Omnibus (GEO) database (https://www.ncbi.nlm.nih.gov/geo/). Finally, two datasets GSE26459 and GSE96570 were selected for screening differential expression genes in TMX-resistant BC, where GSE26459 included 12 control groups and 12 experimental groups, and GSE96570 included 3 control groups and 3 experimental groups.

**FIGURE 1:**
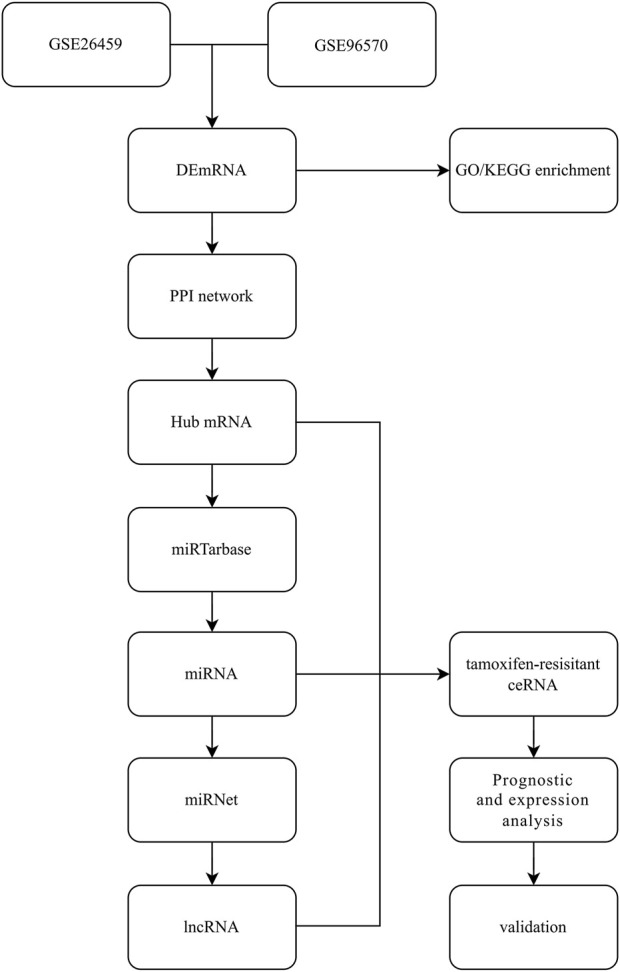
Study design and workflow.

Differential gene analysis was performed on the GEO dataset using GEO2R, setting |Log2FC|>1, *p* < 0.05 as the screening condition, selecting statistically significant results and visualizing the differential genes using volcano plots. The intersection of upregulated and downregulated differential genes was shown using venny plots (https://bioinfogp.cnb.csic.es/tools/venny/index.html). Common differential genes were included in the follow-up study. The workflow is shown in Figure 1.

### GO enrichment and KEGG pathway analysis

To explore the function of known differential genes, Gene Ontology (GO) and KEGG pathway (Kyoto Encyclopedia of Genes and Genomes, KEGG) analyses were conducted on differential mRNAs using the David database (https://david.ncifcrf.gov/). Gene ontology has three main domains: biological processes, cellular components, and molecular functions which can be used to specifically identify the location of gene enrichment, molecular functions and biological processes in an organism. The KEGG database was used to determine the enrichment pathways of key genes and their associated signaling pathways. GO and KEGG enrichment results for key genes were visualized through the bioinformatics website (http://www.bioinformatics.com.cn/).

### PPI network and identification of hub genes

A protein-protein interaction network (PPI) was constructed using the differentially expressed genes in the STRING (https://cn.string-db.org/) database. The multi-protein module was selected on the portal interface, the obtained differential gene set was uploaded, species selection of *Homo sapiens* was performed, and the interaction scores between the genes obtained from the screening were greater than 0.4. After removing isolated genes that do not interact with other proteins, a PPI network map of the target genes was obtained. The degree of association of genes in the PPI network was scored and ranked using the CytoHubba plugin in Cytoscape software (version 3.9.1).

There are some genes in the protein interactions network that are key genes for protein expression; these are involved in certain biological functions of important nodes, which play an important role in the whole protein expression and correspond to the protein interactions network in biology, and are the key genes (hub genes) of the whole PPI. The top 10 highest scoring genes in the upregulated and downregulated networks are displayed as hub genes using CytoHubba’s Betweenness algorithm. These 20 genes were included in the subsequent analysis.

### Prediction of MiRNA and LncRNA

We used MiRTarbase (http://mirtarbase.cuhk.edu.cn/∼miRTarBase/miRTarBase_2022/php/index.php) to predict miRNAs upstream of mRNAs. To achieve more reliable prediction results, we only analyzed mRNA-miRNA interactions that have been experimentally confirmed. MiRTarbase is a database of mRNA-miRNA interactions that have been validated experimentally by protein blotting, microarray and next-generation sequencing.

MiRNet (https://www.mirnet.ca/miRNet/home.xhtml) provides an analysis of miRNA interactions with upstream lncRNAs, allowing the selection of specific tumor tissues to find the corresponding upstream lncRNA target genes. The miRNet database was used to predict upstream lncRNAs of miRNAs by selecting miRNAs from “Organism-Sapiens (human)" “Tissue-Breast cancer tissue”" Target-lncRNAs” to predict the upstream lncRNAs of miRNAs in breast tissue.

### Co-expression analysis

The starBase database (https://starbase.sysu.edu.cn/) has more than 100,000 RNA-seq and 10,000 miRNA-seq data from The Cancer Genome Atlas (TCGA) and Gene Expression Omnibus (GEO) for various types of cancers. We performed gene co-expression analysis using the starBase database to determine the interaction relationships between genes and the strength of the interaction relationships, with *p* < 0.05 being statistically significant. According to the ceRNA hypothesis, lncRNA and mRNA have opposite co-expression relationships with miRNA, while lncRNA has the same co-expression relationship with mRNA. In this study, eligible lncRNAs, mRNAs and miRNAs were selected to construct TMX-resistant ceRNA regulatory networks and visualized by Cytoscape software.

### Survival and expression analysis of tamoxifen resistance genes

The Kaplan–Meier plotter database (http://kmplot.com/) enables the assessment of the survival impact of 54,675 genes in 21 cancers. GEPIA (Gene Expression Profiling Interaction Analysis; http://GEPIA.cancer-pku.cn/detail.php) is a database that integrates TCGA and GEO data to analyze normal and tumor differential expression reliably. The University of Alabama at Birmingham Cancer data analysis portal (UALCAN; http://ualcan.path.uab.edu/analysis.html) can be used to assess the prognosis and expression of all genes and can predict the expression of genes at different stages in cancer. We used the Kaplan–Meier plotter to assess the prognosis of differential genes in breast cancer, GEPIA to assess the expression levels of mRNA and lncRNA, and UALCAN to assess the expression levels of miRNA. We selected genes with consistent prognosis and expression profiles for subsequent experimental validation.

### Cell culture

The human breast cancer MCF-7 cell line used here was obtained from the Shanghai Institute of the Chinese Academy of Sciences. MCF-7 cells were cultured using DMEM complete medium (containing 10% fetal bovine serum; Gibco, Life Technologies, CA, United States) and placed in a 37 °C, 5% CO2 incubator.

### SiRNA and qRT-PCR

A targeted siRNA was designed to knock down SNHG16, and changes in the expression levels of other genes in ceRNA were observed after knocking down this lncRNA. This was used to verify whether drug resistance and prognosis and expression-related ceRNAs in cells hold true (hs-SNHG16-si, F,5′-GCUGCUAAUUGUUCCUCUAAATT-3′, hs-SNHG16-si, R,5′- UUU​AGA​GGA​ACA​AUU​AGC​AGC​TT-3′). We used SYBR-Green to perform qRT-PCR analysis of genes in ceRNA using the comparative CT (2^−ΔΔCT^) method to obtain the relative expression levels of AURKA, has-let-7b-5p and SNHG16 versus GAPDH. The following primer sequences were used: AURKA: F, 5′-GGA​ATA​TGC​ACC​ACT​TGG​AAC​A-3′, R, 5′-GCA​ACC​TCA​GCC​AAG​TAA-3’; has-let-7b-5p: F, 5′-TGT​TGT​AGG​AGC​CCG​TAG-3′, R, 5′-GCA​ACC​TCA​GCC​AAG​TAA-3’; SNHG16: F, 5′-GTT​CCT​CTA​AGT​AAT​CGC​CAT​GCG​TTC​T-3′ R, 5′-CAT​TTC​AGT​TTA​CAA​TCC​TTG​CAG​TCC​C-3’; GAPDH: F, 5′-GCG​AGA​TCG​CAC​TCA​TCA​TCT-3′, R, 5′-TCA​GTG​GTG​GAC​CTG​ACC-3’.

## Results

### Differential gene expression in tamoxifen-resistant breast cancer

**FIGURE 2:**
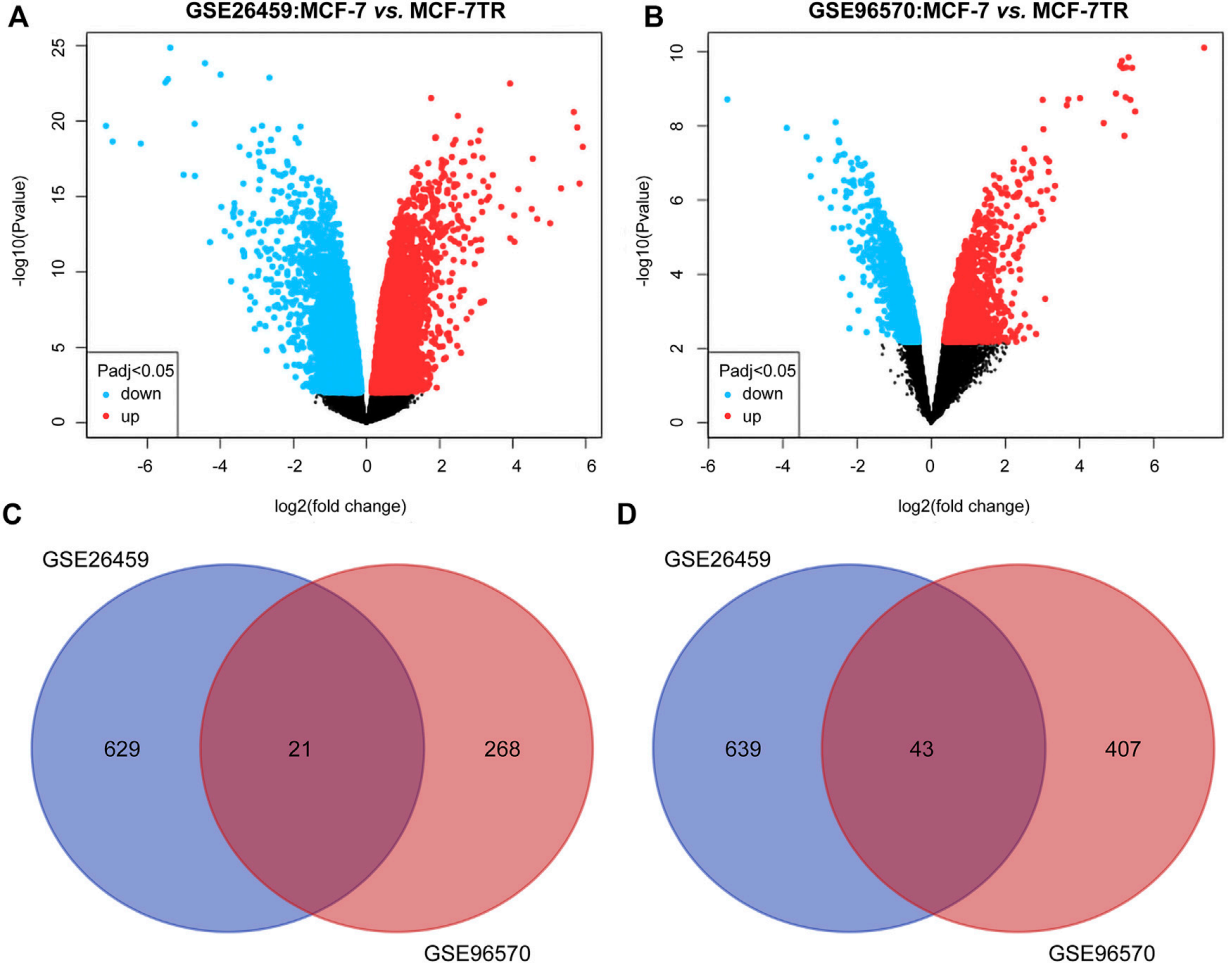
DEmRNAs between TMX-resistant and TMX-sensitive breast cancer in two datasets. **(A**,**B)** Volcano plots of DEmRNAs in GSE26459 and GSE96570. **(C**,**D)** Two datasets intersected by upregulated and downregulated DEmRNAs.

Two TMX-resistant BC datasets (GSE26459, GSE96570) were selected in the GEO database that matched this study. Based on *p* < 0.05 and ∣log2 FC∣>1, 650 upregulated and 682 downregulated mRNAs were screened in the GSE26459 dataset, and 289 upregulated and 450 downregulated mRNAs were screened in the GSE96570 dataset (Figures 2A,B). The intersection of the two mRNA datasets resulted in 21 upregulated and 43 downregulated genes. The co-differentially expressed genes were subjected to subsequent analysis (Figures 2C,D).

### Functional analysis for the DEmRNAs

**FIGURE 3:**
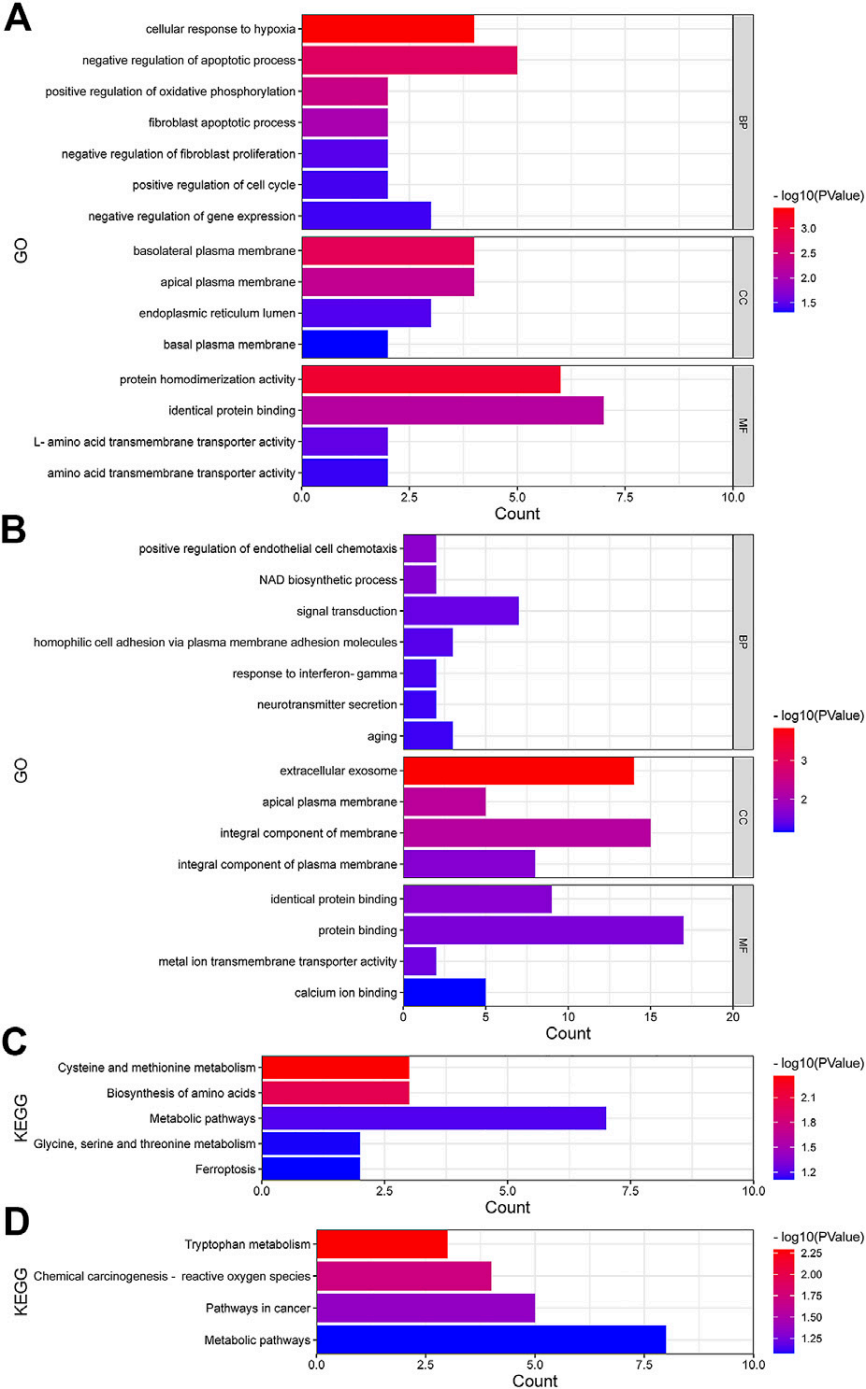
Function analyses in upregulated and downregulated DEmRNAs. **(A**,**B)** GO analysis of upregulated and downregulated DEmRNAs. **(C**,**D)** KEGG pathway analysis of upregulated and downregulated DEmRNAs. BP, biological processes; CC, cellular components; MF, molecular functions.

We performed GO function and KEGG biological pathway enrichment analysis to explore the biological functions of known differential genes. GO functional analysis showed that in biological processes, upregulated differential genes were significantly enriched in the negative regulation of the apoptosis process and the cellular response to hypoxia, and more than half of the downregulated genes were enriched in processes such as signal transduction. In cellular components, upregulated genes were more enriched in basal plasma membrane and apical plasma membrane, while downregulated genes were more enriched in cellular exosomes and basic components of the membrane. In molecular functions, upregulated genes were mostly enriched in protein homogenization activity and identical protein binding, and downregulated genes were mostly enriched in protein binding and identical protein binding (Figures 3A,B). According to the KEGG pathway analysis, most of the upregulated genes were significantly enriched in metabolic pathways (Figures 3C,D), such as cysteine and methionine metabolism and amino acid biosynthesis, while the downregulated genes were enriched in cancer pathways and chemical carcinogen pathways in addition to metabolic pathways. Taken together, these differential genes were enriched in regions of both GO and KEGG that can lead to the development of breast cancer and drug resistance, and the close association with breast cancer suggests that these differential genes have significance in breast cancer research.

### Construction of PPI network and identification of hub genes

**FIGURE 4:**
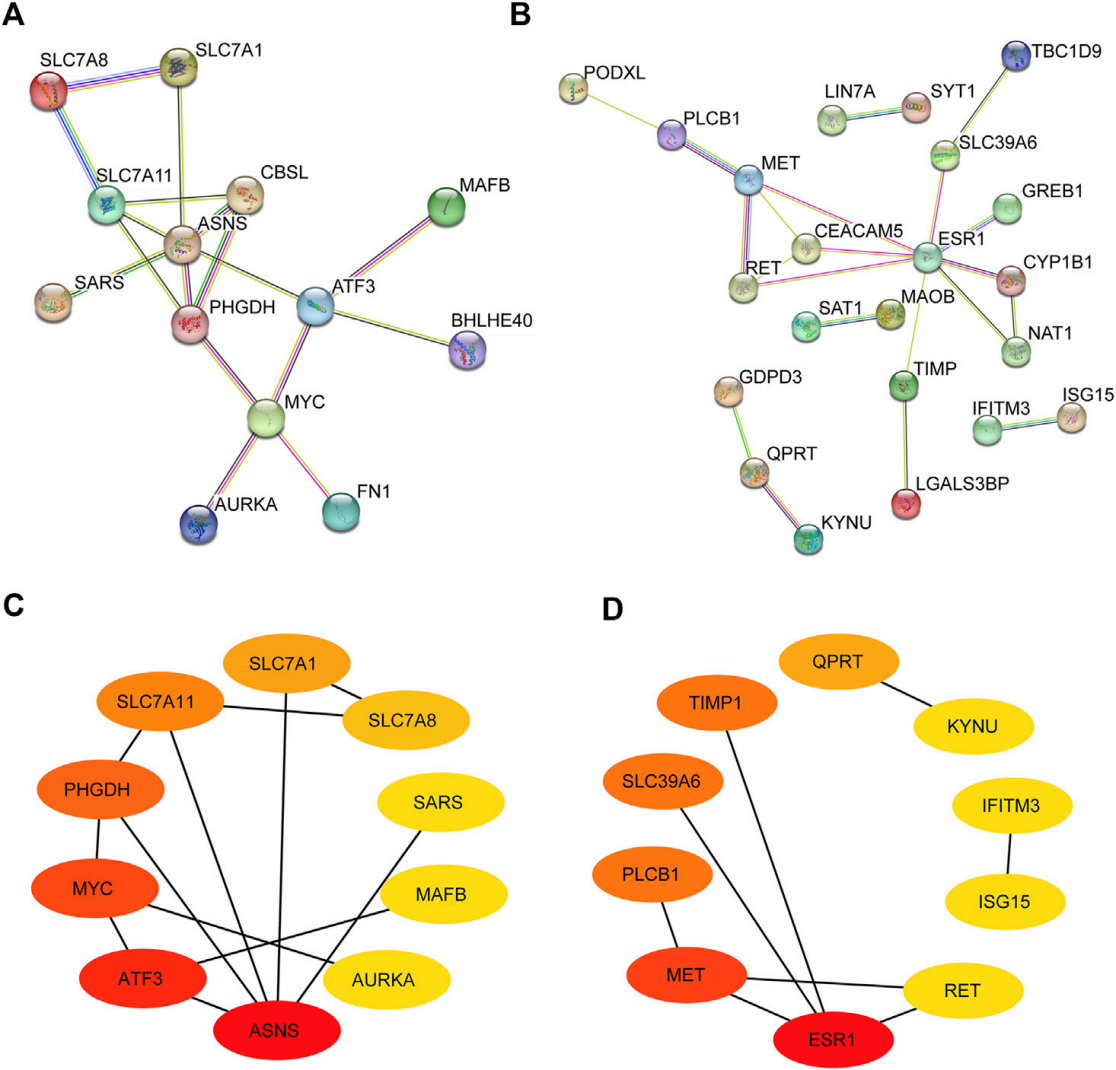
Identification of hub genes in PPI network. **(A**,**B)** PPI network of upregulated and downregulated DEmRNAs. **(C**,**D)** Top 10 upregulated and downregulated hub genes in the PPI network. Darker colors represent higher scores in the network.

To explore the interactions between the screened DEmRNAs, we built a PPI protein interaction network with upregulated and downregulated DEmRNAs, respectively. As shown in Figures 4A,B, there were complex interactions between DEmRNAs, with MYC, ASNS, and SLC7A8 as centers among the upregulated DEmRNAs having larger interaction relationships on surrounding genes; and ESR1 and MET as centers among the downregulated genes having larger interaction relationships on a larger number of surrounding genes. Some of these genes have only a large interaction with another and are not involved in interactions between other genes, such as SAT1 and MAOB, IFITM3 and ASGI5, LIN7A and SYT1, etc. We still consider these genes in the PPI protein interaction network because they also have a strong interaction relationship with each other. Where the gene scores of the PPI network were obtained by calculating the scores based on the cytoHubba plugin in Cytoscape software, we selected the top 10 gene scores in the upregulated and downregulated PPI networks as the key nodes (Figures 4C,D). The top 10 upregulated key genes were ASNS, ATF3, MYC, PHGDH, SLC7A11, SLC7A1, SLC7A8, SARS, MAFB, and AURKA. The top 10 downregulated key genes were ESR1, MET, PLCB1, SLC39A6, TIMP1, QPRT, KYNU, IFITM3, ISG15, and RET. These 20 genes will be further analyzed later.

### Prediction of upstream MiRNA and LncRNA

MiRTarbase and miRNet databases were used to predict miRNAs and lncRNAs upstream of 20 hub genes. To enhance the confidence of the prediction results, we chose miRNA-mRNA, miRNA-lncRNA and mRNA-lncRNA interactions that were experimentally confirmed in previous articles. After screening the database, a total of 113 upstream miRNAs were determined for 8 key genes, with 64 miRNAs corresponding to 4 upregulated genes (SLC7A1, PHGDH, AURKA and ASNS) and 49 miRNAs corresponding to 4 downregulated genes (TIMP1, SLC39A6, ESR1 and RET).

All miRNAs correspond to a total of 501 upstream lncRNAs, of which 7 downregulated miRNAs (hsa-let-7b-5p, has-101-3p, hsa-mir-10a-5p, has-mir-125b-5p, has-mir-29a-3p, has-mir-376a-3p and has -mir-130a-3p) corresponded to 336 upstream lncRNAs, and there were 4 upregulated miRNAs (hsa-mir-19b-3p, hsa-mir-21-5p, hsa-mir-17-5p and hsa-mir-210-3p) corresponding to 165 upstream lncRNAs.

### Establishment of ceRNA regulatory network for TMX resistance in BC

**FIGURE 5:**
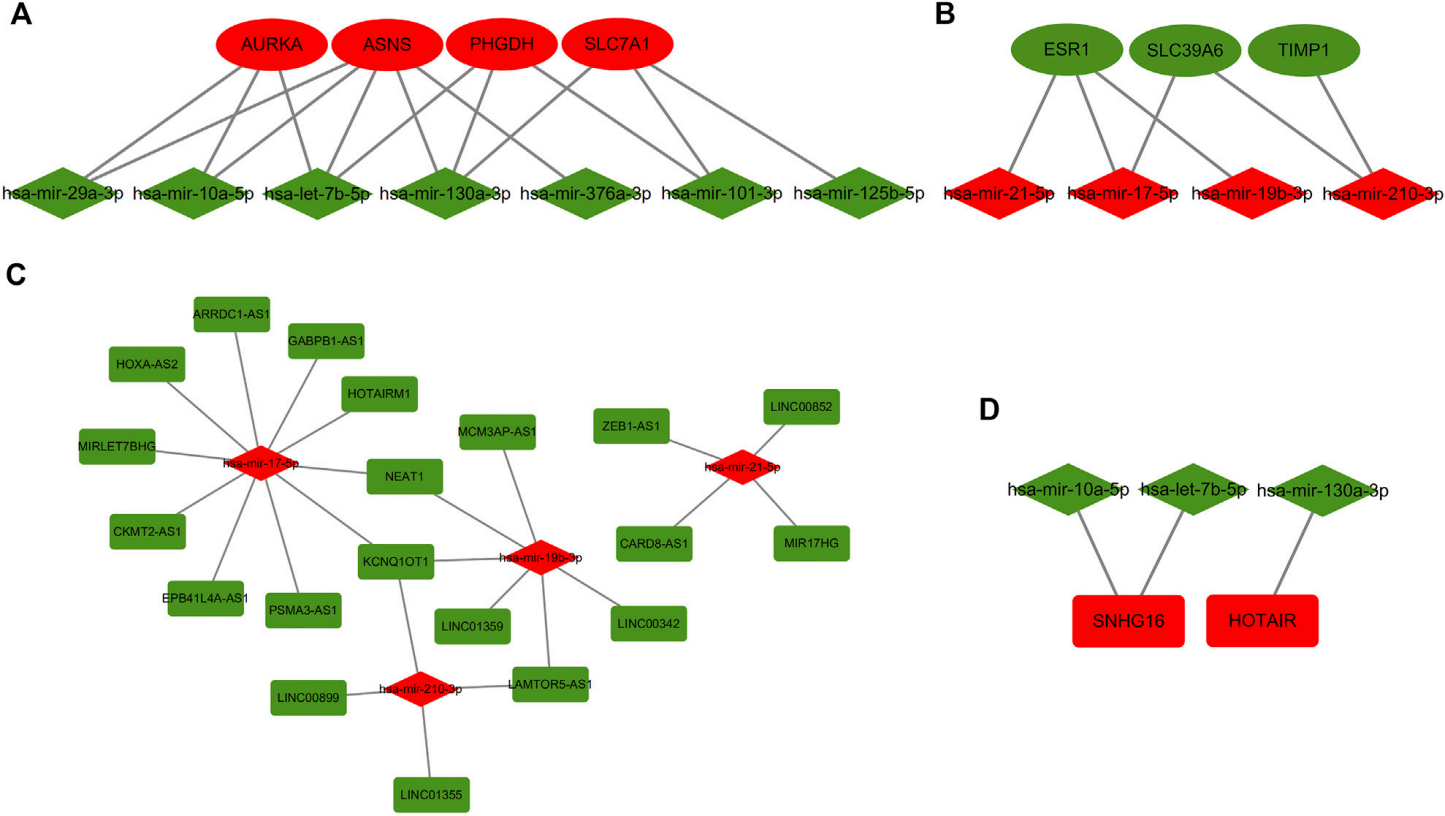
Regulatory network of miRNA-mRNA and miRNA-lncRNA for TMX-resistant breast cancer. **(A**,**B)** mRNA-miRNA correspondence. **(C**,**D)** miRNA-lncRNA correspondence. Oval shape represents mRNA, diamond shape represents miRNA, and rectangle shape represents lncRNA. Red color represents upregulated genes and green color represents downregulated genes.

The ceRNA hypothesis suggests that lncRNAs compete as sponges for binding to miRNAs(Thomson and Dinger, 2016). LncRNA expression levels should have a negative correlation with miRNAs and a positive correlation with mRNA. According to the results of the starBase database, there were 20 mRNA-miRNA pairs (Figures 5A,B) and 27 miRNA-lncRNA pairs (Figures 5C,D). All pairwise relationships were visualized by Cytoscape software. Each component of these pairings was oppositely expressed. Finally, we identified co-expression relationships between 7 mRNAs, 7 miRNAs and 22 lncRNAs, consistent with the rules of the ceRNA hypothesis, and constructed the BC TMX resistance ceRNA regulatory network accordingly.

### Prognostic and expression analysis of genes in TMX-Resistant BC ceRNA

**FIGURE 6:**
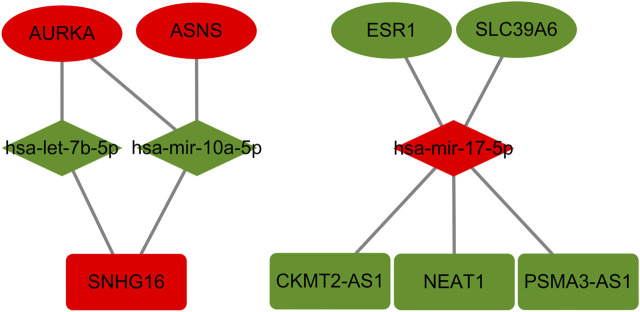
Prognosis-related genes in TMX-resistant BC. Ovals in the figure represent mRNAs, red ovals represent upregulated mRNAs and green ovals represent downregulated mRNAs; the diamonds represent miRNAs, red diamonds represent upregulated miRNAs and green diamonds represent downregulated miRNAs; the rectangles represent lncRNAs, red rectangles represent upregulated lncRNAs and green rectangles represent downregulated lncRNAs.

To obtain prognosis- and expression-related genes in the TMX-resistant BC ceRNA regulatory network, we assessed the core gene expression and prognostic relationships using the GEPIA and Kaplan–Meier databases. Of these differentially expressed genes, both prognosis and expression had to meet the same correspondence. We obtained 4 mRNAs, 3 miRNAs, and 4 mRNAs, of which AURKA, ASNS, hsa-mir-17-5p and SNHG16 expression was upregulated, and ESR1, SLC39A6, hsa-let-7b-5p, hsa-mir-10a-5p, CKMT2-AS1 and PSAM3-AS1 expression was downregulated (Figure 6). These genes are differentially expressed genes in drug-resistant BC and are also associated with BC prognosis. We listed the strength of the interactions between these genes according to the starBase database (Table 1). All correlations had a *p*-value < 0.05.

**TABLE 1:** The correlation between miRNA-mRNA, miRNA-lncRNA and mRNA-lncRNA according to the starBase database. R represents the correlation coefficient between genes and all *p* values are less than 0.05.

miRNA/mRNA	mRNA/lncRNA	R
has-let-7b-5p	AURKA	−0.297
has-mir-10a-5p	AURKA	−0.273
hsa-mir-10a-5p	ASNS	−0.241
has-mir-17-5p	ESR1	−0.486
has-mir-17-5p	SLC39A6	−0.371
has-let-7b-5p	SNHG16	−0.149
has-mir-10a-5p	SNHG16	−0.176
has-mir-17-5p	CKMT2-AS1	−0.328
has-mir-17-5p	NEAT1	−0.257
has-mir-17-5p	PSMA3-AS1	−0.258
AURKA	SNHG16	0.38
ASNS	SNHG16	0.243
ESR1	CKMT-AS1	0.383
ESR1	NEAT1	0.382
ESR1	PSMA3-AS1	0.473
SLC39A6	CKMT2-AS1	0.283
SLC39A6	NEAT1	0.238
SLC39A6	PSMA3-AS1	0.305

**FIGURE 7:**
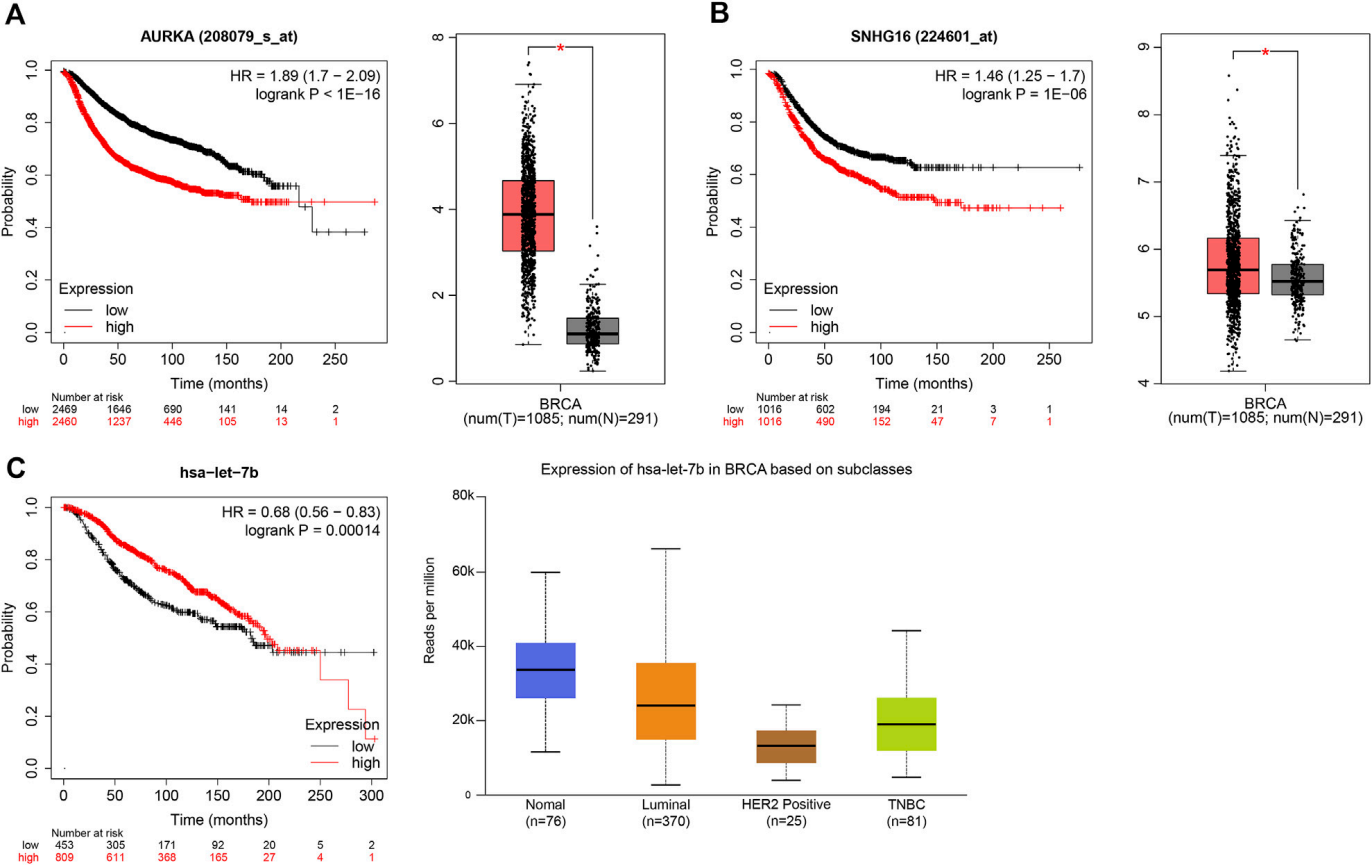
Prognosis and expression of AURKA, has-let-7b-5p and SNHG16. **(A)** The prognosis and expression of AURKA in Kaplan–Meier and GEPIA. **(B)**The prognosis and expression of SNHG16 in Kaplan–Meier and GEPIA. **(C)** The prognosis and expression of has-let-7b-5p in Kaplan–Meier and UALCAN.

Overall, there was a strong correlation between the AURKA-has-let-7b-5p-SNHG16 axis, and each gene was strongly correlated in BC prognosis and expression according to Kaplan–Meier, GEPIA and UALCAN analyses (Figure 7). All three genes have a large literature demonstrating their greater role in BC prognosis and drug resistance (Peng et al., 2021); (Wander et al., 2020); (Ni et al., 2020) (Lirussi et al., 2022). We finally selected the sub-network AURKA-has-let-7b-5p-SNHG16 for analysis and validation.

### LncRNA SNHG16 affects gene expression in the AURKA-hsa-let-7b-5p-SNHG16 axis

**FIGURE 8:**
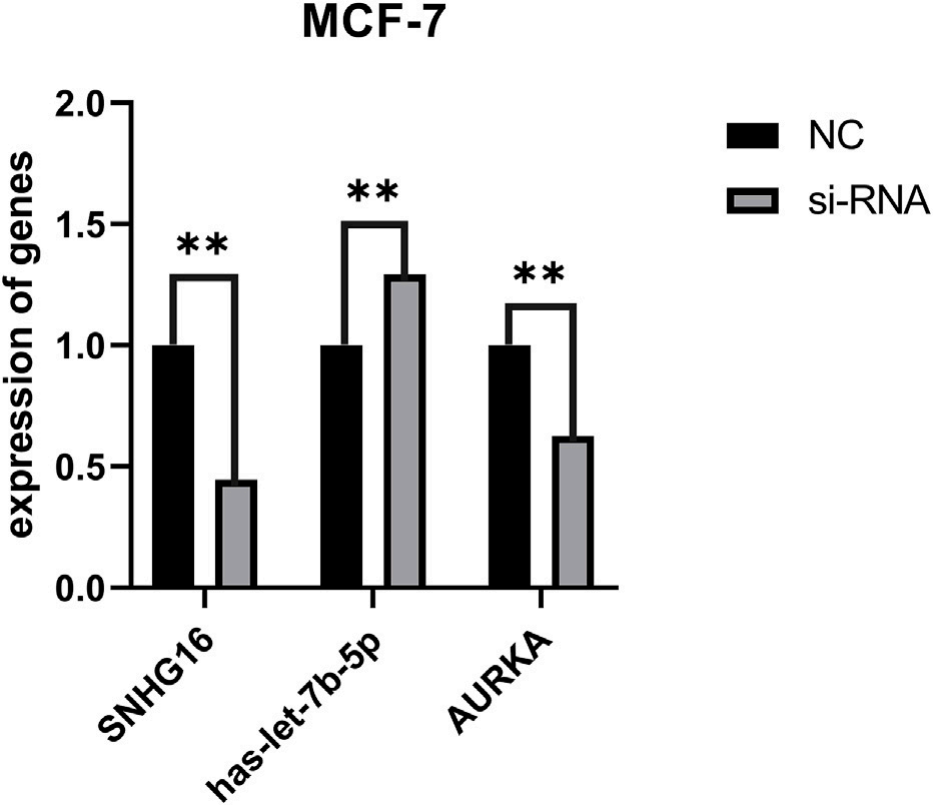
Changes in ceRNA genes after knockdown of SNHG16. The horizontal coordinates represent SNHG16, hesa-let-7b-5p and AURKA before and after transfection, respectively. The vertical coordinates represent gene expression; black represents the expression of control genes and grey represents the expression of genes after transfection. **p < 0.01.

Through bioinformatic analysis, we found that SNHG16-has-let-7b-5p-AURKA axis is present in BC and plays a role in drug resistance. We selected MCF-7 cells to validate the intracellular interactions of this ceRNA network to further improve the reliability of the results. We designed siRNA to knock down lncRNA SNHG16 and validated the knockdown efficiency using qRT-PCR *in vitro*. SNHG16 acts as an upstream lncRNA in ceRNA, and according to the ceRNA hypothesis, when SNHG16 is knocked down, the expression of has-let-7b-5p in cells should subsequently decrease and the expression of AURKA should increase. The changes in miRNA and mRNA expression when SNHG16 was knocked down compared to controls were fully consistent with the ceRNA hypothesis (Figure 8), suggesting that the SNHG16-has-let-7b-5p-AURKA axis is present in BC, belongs to the ceRNA regulatory network, and plays an important role in BC prognosis and drug resistance, with potential to become a biomarker in the future.

## Discussion

BC is the most commonly diagnosed cancer in women and endangers the health of women worldwide (Siegel et al., 2022). ER-positive BC accounts for the largest proportion of breast cancer staging, approximately two-thirds (Ignatiadis and Sotiriou, 2013). With the development of pharmaceutical technology, endocrine therapy (e.g., TMX) has become the most effective means of curing ER-positive BC (Early Breast Cancer Trialists’ Collaborative Group (EBCTCG), 2022), but drug resistance developed during treatment remains the greatest challenge threatening patient survival (Gong et al., 2018). Therefore, it is very important to reveal the molecular switches that generate resistance in ER-positive BC treatment, which also helps to uncover new prognostic indicators.

It is well known that lncRNAs play an increasingly important role in the development of cancer (Li et al., 2017; Peng et al., 2017). Karreth et al. pointed out that lncRNAs can act as molecular sponges for miRNAs to regulate the activity and function of mRNAs, and lncRNAs can act as an important post-transcriptional regulators of downstream gene expression through miRNA-mediated mechanisms (Karreth and Pandolfi, 2013). The hypothesis of ceRNAs was thus proposed. Since then, an increasing number of studies have shown that ceRNAs are involved in various pathological processes including tumorigenesis (Qi et al., 2015). Zhang et al. reported that lncRNA RP11-1094M14.8 as ceRNA regulates miR-1269a downstream target gene CXCL9 to promote the development of gastric cancer (Zhang et al., 2020); Luan et al. noted that when XLOC_006390 was knocked down, the expression of miR-331-3p downstream gene NRP2 was downregulated, and the expression of miR-338-3p downstream genes PKM2 and EYA2 was also downregulated, thus inhibiting cervical carcinogenesis and metastasis (Luan and Wang, 2018); Zhou et al. pointed out that multiple lncRNAs (LINC02560, HOXC13 - AS, FOXD2 - AS1, etc.) acted as ceRNAs to bind to downstream miRNA loci to regulate mRNA expression and influence the progression of squamous cell carcinoma of the tongue (Zhou et al., 2019). In addition, many new evidences suggest that ceRNA plays an important role in BC, for example, lncRNA-CDC6 as ceRNA regulates the expression of CDC6 by sponging on microRNA-215, thus promoting metastasis and proliferation of BC (Kong et al., 2019). ceRNA also plays an important role in ER-positive BC, the largest proportion of BC staging. Wang et al. showed that circPGR functions as a ceRNA to regulate the expression of several cell cycle genes which can effectively inhibit the growth of ER-positive BC cells (Wang et al., 2021b).

Recent research suggests that ceRNAs are also involved in drug resistance in BC, influencing cancer drug sensitivity through various intracellular biological processes (Liu et al., 2021). Sang et al. showed that circRNA_0025202, a ceRNA, inhibits TMX resistance in MCF-7 cells by regulating the miR-182-5p/FOXO3a axis in BC, thereby inhibiting tumor development (Sang et al., 2021). Dong et al. showed that the lncRNA, SNHG14, regulates PABPC1 expression through H3K27 acetylation and induces resistance to trastuzumab in BC (Dong et al., 2018).

In our study we first constructed a ceRNA regulatory network for TMX-resistant BC, and then analyzed the core genes in the ceRNA network to screen out the genes associated with prognosis and expression of BC. These genes were not only involved in drug resistance of BC, but also associated with BC prognosis. Using this ceRNA as a prediction model for BC drug-resistance largely improved the accuracy and stability of prediction.

First, we identified two TMX resistance GEO datasets (GSE26459 and GSE96570). We screened 21 upregulated differential genes and 43 downregulated differential genes by comparing drug-resistant BC tissues with normal BC tissues. We performed functional enrichment analysis of these differential genes and GO analysis showed that most genes were enriched in cancer-related functions, such as negative regulation of apoptosis (Thrane et al., 2015), cellular response to hypoxia (Whately et al., 2021), and protein homogenization activity (Skidmore et al., 2020); these processes were also closely associated with drug resistance in BC. In the KEGG analysis, differential genes were mostly enriched in metabolic pathways and cancer-related pathways (Erdelyi et al., 2021).

To explore the interactions between these differential genes, we constructed PPI networks in the STRING database for upregulated and downregulated genes, and showed a large correlation between these genes. We then calculated the nodes with high correlation scores in cytoscape software and selected the top 10 scored nodes as hub genes in the upregulated and downregulated PPI networks. A simple search for these genes surprisingly revealed that almost all genes were widely reported in cancer, and some even reported in drug-resistant BC. For example, ASNS has a strong correlation in cancers such as ovarian cancer (Zeng et al., 2019), leukemia (Watanabe et al., 2020) and B-cell lymphoma (Grima-Reyes et al., 2022). AURKA and ESR1 confer cellular drug resistance when overexpressed in ER-positive BC (Wander et al., 2020; Herzog and Fuqua, 2022). This proves that the key genes obtained from our screen are very reliable and may become key genes for BC development and drug-resistant BC.

To construct a BC drug resistance ceRNA regulatory network, we used MiRTarbase and miRNet to successively obtain upstream miRNAs and lncRNAs of hub genes. Based on the ceRNA hypothesis, we constructed a ceRNA regulatory network for TMX-resistant BC using 7 mRNAs, 7 miRNAs and 22 lncRNAs.

We also determined the expression and prognosis of genes in drug-resistant ceRNAs in BC. Genes consistent with BC prognosis and expression were selected. We finally identified 4 mRNAs (AURKA, ASNS, ESR1 and SLC39A6), 3 miRNAs (has-let-7b-5p, has-mir-10a-5p and has-mir-17-5p), and 4 lncRNAs (SNHG16, CKMT2-AS1, NEAT1 and PSMA3-AS1), all of which have been reported in cancer. For example, downregulation of has-let-7b-5p enhances XIST expression leading to cisplatin resistance in gastric cancer (Han et al., 2020; Rong et al., 2020). More importantly, it has been shown that has-let-7b induces TMX sensitivity in BC through downregulation of ER-α signaling (Zhao et al., 2011). SNHG16 is overexpressed in a variety of cancers (Gong et al., 2020), such as neuroblastoma (Xu et al., 2020), lung cancer (Xia et al., 2021), thyroid cancer (Wen et al., 2019), and breast cancer (Du et al., 2020). SNHG16 also plays a role in tumor drug resistance. In neuroblastoma cells, SNHG16 can act as a molecular sponge for miR-338-3p to increase PLK4 expression, which in turn promotes tumor development and enhances cisplatin resistance (Xu et al., 2020). Ye et al. demonstrated that lncRNA, SNHG16, induces drug resistance in hepatocellular carcinoma cells by sponging has-miR-140-5p (Ye et al., 2019). We successfully constructed a new ceRNA that correlates with drug resistance in BC, and interestingly found that some of the interactions in this network have been reported in previous articles. Li et al. found that SNHG16, a sponge of miRNA has-let-7b-5p, induced drug resistance in hepatocellular carcinoma by upregulating the expression of CDC25B and HMGA2, and that downregulation of SNHG16 led to apoptosis (Li et al., 2020). NEAT1 regulates angiogenesis and promotes gastric cancer progression through upregulation of the miR-17-5p/TGFβR2 axis (Xu et al., 2021). This data further indicates that the ceRNA we have constructed is reliable. Finally, we performed co-expression analysis of these genes by starBase, and finally selected a sub-network (SNHG16-has-let-7b-5p-AURKA) that was more correlated and consistent with the ceRNA hypothesis for experimental validation. The results are fully consistent with our previous analysis.

Inevitably, there are some consideration flaws and limitations in our study. Firstly, in constructing the ceRNA regulatory network, we considered both the hypothesis of ceRNAs and the prognostic value of genes, which overlooked many potentially valuable genes. Secondly, we did not analyze the differences in the development of TMX-resistant BC of different stages and different ages. For example, conservative treatment would be chosen in early treatment of breast cancer, and there would be differences in treatment for BC before and after menopause. Thirdly, there is usually more than one drug used in the clinical treatment of BC. We did not analyze the effect of combination drugs on drug resistance in BC treatment.

In conclusion, we successfully constructed a drug resistance ceRNA regulatory network in breast cancer. We also screened for, and validated, genes associated with BC prognosis. This may lead to new treatment strategies for TMX-resistant BC and may also provide new ideas in the clinical treatment of drug resistance.

## Data Availability

The datasets presented in this study can be found in online repositories. The names of the repository/repositories and accession number(s) can be found in the article/Supplementary Material.

## References

[B1] Asif H. M. Sultana S. Ahmed S. Akhtar N. Tariq M. (2016). HER-2 positive breast cancer - a mini-review. Asian pac. J. Cancer Prev. 17 (4), 1609–1615. 10.7314/apjcp.2016.17.4.1609 27221828

[B2] Behera J. Kumar A. Voor M. J. Tyagi N. (2021). Exosomal lncRNA-H19 promotes osteogenesis and angiogenesis through mediating Angpt1/Tie2-NO signaling in CBS-heterozygous mice. Theranostics 11 (16), 7715–7734. 10.7150/thno.58410 34335960 PMC8315071

[B4] Dong H. Wang W. Mo S. Liu Q. Chen X. Chen R. (2018). Long non-coding RNA SNHG14 induces trastuzumab resistance of breast cancer via regulating PABPC1 expression through H3K27 acetylation. J. Cell. Mol. Med. 22 (10), 4935–4947. 10.1111/jcmm.13758 30063126 PMC6156344

[B5] Du C. Wang Y. Zhang Y. Zhang J. Zhang L. Li J. (2020). LncRNA DLX6-AS1 contributes to epithelial-mesenchymal transition and cisplatin resistance in triple-negative breast cancer via modulating mir-199b-5p/paxillin Axis. Cell Transpl. 29, 963689720929983. 10.1177/0963689720929983 PMC7563824 32686982

[B6] Du S. M. (2020). The SNHG16/miR-30a axis promotes breast cancer cell proliferation and invasion by regulating RRM2. Neoplasma 67 (3), 567–575. 10.4149/neo_2020_190625N550 32122142

[B8] Early Breast Cancer Trialists’ Collaborative Group (EBCTCG) (2022). Aromatase inhibitors versus tamoxifen in premenopausal women with oestrogen receptor-positive early-stage breast cancer treated with ovarian suppression: A patient-level meta-analysis of 7030 women from four randomised trials. Lancet. Oncol. 23 (3), 382–392. 10.1016/S1470-2045(21)00758-0 35123662 PMC8885431

[B9] Erdelyi K. Ditroi T. Johansson H. J. Czikora A. Balog N. Silwal-Pandit L. (2021). Reprogrammed transsulfuration promotes basal-like breast tumor progression via realigning cellular cysteine persulfidation. Proc. Natl. Acad. Sci. U. S. A. 118 (45), e2100050118. 10.1073/pnas.2100050118 34737229 PMC8609449

[B10] Gong C. Man E. Tsoi H. Lee T. Lee P. Ma S. T. (2018). BQ323636.1, a novel splice variant to NCOR2, as a predictor for tamoxifen-resistant breast cancer. Clin. Cancer Res. 24 (15), 3681–3691. 10.1158/1078-0432.CCR-17-2259 29420220 PMC6038915

[B11] Gong C. Y. Tang R. Nan W. Zhou K. S. Zhang H. H. (2020). Role of SNHG16 in human cancer. Clin. Chim. Acta. 503, 175–180. 10.1016/j.cca.2019.12.023 31901482

[B12] Grima-Reyes M. Vandenberghe A. Nemazanyy I. Meola P. Paul R. Reverso-Meinietti J. (2022). Tumoral microenvironment prevents de novo asparagine biosynthesis in B cell lymphoma, regardless of ASNS expression. Sci. Adv. 8 (27), n6491. 10.1126/sciadv.abn6491 PMC9258813 35857457

[B13] Han X. Zhang H. B. Li X. D. Wang Z. A. (2020). Long non-coding RNA X-inactive-specific transcript contributes to cisplatin resistance in gastric cancer by sponging miR-let-7b. Anticancer. Drugs 31 (10), 1018–1025. 10.1097/CAD.0000000000000942 33009035

[B14] Herzog S. K. Fuqua S. (2022). ESR1 mutations and therapeutic resistance in metastatic breast cancer: Progress and remaining challenges. Br. J. Cancer 126 (2), 174–186. 10.1038/s41416-021-01564-x 34621045 PMC8770568

[B15] Ignatiadis M. Sotiriou C. (2013). Luminal breast cancer: From biology to treatment. Nat. Rev. Clin. Oncol. 10 (9), 494–506. 10.1038/nrclinonc.2013.124 23881035

[B16] Jia X. Hong Q. Lei L. Li D. Li J. Mo M. (2015). Basal and therapy-driven hypoxia-inducible factor-1α confers resistance to endocrine therapy in estrogen receptor-positive breast cancer. Oncotarget 6 (11), 8648–8662. 10.18632/oncotarget.3257 25929338 PMC4496173

[B17] Jiang R. Zhao C. Gao B. Xu J. Song W. Shi P. (2018). Mixomics analysis of breast cancer: Long non-coding RNA linc01561 acts as ceRNA involved in the progression of breast cancer. Int. J. Biochem. Cell Biol. 102, 1–9. 10.1016/j.biocel.2018.06.003 29890225

[B18] Karreth F. A. Pandolfi P. P. (2013). ceRNA cross-talk in cancer: when ce-bling rivalries go awry. Cancer Discov. 3 (10), 1113–1121. 10.1158/2159-8290.CD-13-0202 24072616 PMC3801300

[B19] Kong X. Duan Y. Sang Y. Li Y. Zhang H. Liang Y. (2019). LncRNA-CDC6 promotes breast cancer progression and function as ceRNA to target CDC6 by sponging microRNA-215. J. Cell. Physiol. 234 (6), 9105–9117. 10.1002/jcp.27587 30362551

[B20] Lambert L. K. Balneaves L. G. Howard A. F. Chia S. K. Gotay C. C. (2018). Understanding adjuvant endocrine therapy persistence in breast Cancer survivors. BMC Cancer 18 (1), 732. 10.1186/s12885-018-4644-7 29996816 PMC6042363

[B21] Li J. Ke J. Qin C. L. Zhu X. (2022). LINC00680 modulates docetaxel resistance in breast cancer via the miR-320b/CDKL5 axis. Int. J. Immunopathol. Pharmacol. 36, 3946320221105608. 10.1177/03946320221105608 35667653 PMC9178731

[B22] Li S. Peng F. Ning Y. Jiang P. Peng J. Ding X. (2020). SNHG16 as the miRNA let-7b-5p sponge facilitates the G2/M and epithelial-mesenchymal transition by regulating CDC25B and HMGA2 expression in hepatocellular carcinoma. J. Cell. Biochem. 121 (3), 2543–2558. 10.1002/jcb.29477 31696971

[B23] Li W. Zhang Z. Liu X. Cheng X. Zhang Y. Han X. (2017). The FOXN3-NEAT1-SIN3A repressor complex promotes progression of hormonally responsive breast cancer. J. Clin. Invest. 127 (9), 3421–3440. 10.1172/JCI94233 28805661 PMC5669564

[B24] Lirussi L. Ayyildiz D. Liu Y. Montaldo N. P. Carracedo S. Aure M. R. (2022). A regulatory network comprising let-7 miRNA and SMUG1 is associated with good prognosis in ER+ breast tumours. Nucleic Acids Res. 50, 10449–10468. 10.1093/nar/gkac807 36156150 PMC9561369

[B25] Liu B. Zhou X. Wu D. Zhang X. Shen X. Mi K. (2021). Comprehensive characterization of a drug-resistance-related ceRNA network across 15 anti-cancer drug categories. Mol. Ther. Nucleic Acids 24, 11–24. 10.1016/j.omtn.2021.02.011 33738135 PMC7933708

[B26] Luan X. Wang Y. (2018). LncRNA XLOC_006390 facilitates cervical cancer tumorigenesis and metastasis as a ceRNA against miR-331-3p and miR-338-3p. J. Gynecol. Oncol. 29 (6), e95. 10.3802/jgo.2018.29.e95 30207103 PMC6189437

[B27] Natarajan K. Xie Y. Baer M. R. Ross D. D. (2012). Role of breast cancer resistance protein (BCRP/ABCG2) in cancer drug resistance. Biochem. Pharmacol. 83 (8), 1084–1103. 10.1016/j.bcp.2012.01.002 22248732 PMC3307098

[B28] Ni C. Fang Q. Q. Chen W. Z. Jiang J. X. Jiang Z. Ye J. (2020). Breast cancer-derived exosomes transmit lncRNA SNHG16 to induce CD73+γδ1 Treg cells. Signal Transduct. Target. Ther. 5 (1), 41. 10.1038/s41392-020-0129-7 32345959 PMC7188864

[B29] Peng F. Xu J. Cui B. Liang Q. Zeng S. He B. (2021). Oncogenic AURKA-enhanced N(6)-methyladenosine modification increases DROSHA mRNA stability to transactivate STC1 in breast cancer stem-like cells. Cell Res. 31 (3), 345–361. 10.1038/s41422-020-00397-2 32859993 PMC8027457

[B30] Peng W. X. Huang J. G. Yang L. Gong A. H. Mo Y. Y. (2017). Linc-RoR promotes MAPK/ERK signaling and confers estrogen-independent growth of breast cancer. Mol. Cancer 16 (1), 161. 10.1186/s12943-017-0727-3 29041978 PMC5645922

[B31] Qi X. Zhang D. H. Wu N. Xiao J. H. Wang X. Ma W. (2015). ceRNA in cancer: possible functions and clinical implications. J. Med. Genet. 52 (10), 710–718. 10.1136/jmedgenet-2015-103334 26358722

[B32] Rong J. Xu L. Hu Y. Liu F. Yu Y. Guo H. (2020). Inhibition of let-7b-5p contributes to an anti-tumorigenic macrophage phenotype through the SOCS1/STAT pathway in prostate cancer. Cancer Cell Int. 20, 470. 10.1186/s12935-020-01563-7 33005103 PMC7526222

[B33] Salmena L. Poliseno L. Tay Y. Kats L. Pandolfi P. P. (2011). A ceRNA hypothesis: The rosetta stone of a hidden RNA language? Cell 146 (3), 353–358. 10.1016/j.cell.2011.07.014 21802130 PMC3235919

[B34] Sang Y. Chen B. Song X. Li Y. Liang Y. Han D. (2021). circRNA_0025202 regulates tamoxifen sensitivity and tumor progression via regulating the miR-182-5p/FOXO3a Axis in breast cancer. Mol. Ther. 29 (12), 3525–3527. 10.1016/j.ymthe.2021.11.002 34774125 PMC8636162

[B35] Siegel R. L. Miller K. D. Fuchs H. E. Jemal A. (2022). Cancer statistics, 2022. Ca. Cancer J. Clin. 72 (1), 7–33. 10.3322/caac.21708 35020204

[B36] Skidmore L. Sakamuri S. Knudsen N. A. Hewet A. G. Milutinovic S. Barkho W. (2020). ARX788, a site-specific anti-HER2 antibody-drug conjugate, demonstrates potent and selective activity in HER2-low and T-DM1-resistant breast and gastric cancers. Mol. Cancer Ther. 19 (9), 1833–1843. 10.1158/1535-7163.MCT-19-1004 32669315

[B37] Tang W. Zhang X. Tan W. Gao J. Pan L. Ye X. (2019). miR-145-5p suppresses breast cancer progression by inhibiting SOX2. J. Surg. Res. 236, 278–287. 10.1016/j.jss.2018.11.030 30694767

[B38] Thomson D. W. Dinger M. E. (2016). Endogenous microRNA sponges: Evidence and controversy. Nat. Rev. Genet. 17 (5), 272–283. 10.1038/nrg.2016.20 27040487

[B39] Thrane S. Pedersen A. M. Thomsen M. B. Kirkegaard T. Rasmussen B. B. Duun-Henriksen A. K. (2015). A kinase inhibitor screen identifies Mcl-1 and Aurora kinase A as novel treatment targets in antiestrogen-resistant breast cancer cells. Oncogene 34 (32), 4199–4210. 10.1038/onc.2014.351 25362855

[B40] Wander S. A. Cohen O. Gong X. Johnson G. N. Buendia-Buendia J. E. Lloyd M. R. (2020). The genomic landscape of intrinsic and acquired resistance to cyclin-dependent kinase 4/6 inhibitors in patients with hormone receptor-positive metastatic breast cancer. Cancer Discov. 10 (8), 1174–1193. 10.1158/2159-8290.CD-19-1390 32404308 PMC8815415

[B41] Wang L. Yi J. Lu L. Y. Zhang Y. Y. Wang L. Hu G. S. (2021a). Estrogen-induced circRNA, circPGR, functions as a ceRNA to promote estrogen receptor-positive breast cancer cell growth by regulating cell cycle-related genes. Theranostics 11 (4), 1732–1752. 10.7150/thno.45302 33408778 PMC7778588

[B42] Wang L. Zhou Y. Jiang L. Lu L. Dai T. Li A. (2021b). CircWAC induces chemotherapeutic resistance in triple-negative breast cancer by targeting miR-142, upregulating WWP1 and activating the PI3K/AKT pathway. Mol. Cancer 20 (1), 43. 10.1186/s12943-021-01332-8 33648498 PMC7919093

[B43] Watanabe A. Miyake K. Nordlund J. Syvanen A. C. van der Weyden L. Honda H. (2020). Association of aberrant ASNS imprinting with asparaginase sensitivity and chromosomal abnormality in childhood BCP-ALL. Blood 136 (20), 2319–2333. 10.1182/blood.2019004090 32573712 PMC7702480

[B44] Wen Q. Zhao L. Wang T. Lv N. Cheng X. Zhang G. (2019). LncRNA SNHG16 drives proliferation and invasion of papillary thyroid cancer through modulation of miR-497. Onco. Targets. Ther. 12, 699–708. 10.2147/OTT.S186923 30705598 PMC6343509

[B45] Whately K. M. Voronkova M. A. Maskey A. Gandhi J. Loskutov J. Choi H. (2021). Nuclear aurora-A kinase-induced hypoxia signaling drives early dissemination and metastasis in breast cancer: Implications for detection of metastatic tumors. Oncogene 40 (37), 5651–5664. 10.1038/s41388-021-01969-1 34326467 PMC9511212

[B46] Xia W. Liu Y. Cheng T. Xu T. Dong M. Hu X. (2021). Extracellular vesicles carry lncRNA SNHG16 to promote metastasis of breast cancer cells via the miR-892b/ppapdc1a Axis. Front. Cell Dev. Biol. 9, 628573. 10.3389/fcell.2021.628573 34249903 PMC8267525

[B47] Xu Y. Li Y. Qiu Y. Sun F. Zhu G. Sun J. (2021). LncRNA NEAT1 promotes gastric cancer progression through miR-17-5p/tgfßr2 Axis up-regulated angiogenesis. Front. Cell Dev. Biol. 9, 705697. 10.3389/fcell.2021.705697 34552925 PMC8452045

[B48] Xu Z. Sun Y. Wang D. Sun H. Liu X. (2020). SNHG16 promotes tumorigenesis and cisplatin resistance by regulating miR-338-3p/PLK4 pathway in neuroblastoma cells. Cancer Cell Int. 20, 236. 10.1186/s12935-020-01291-y 32536824 PMC7291484

[B49] Yan L. Yang S. Yue C. X. Wei X. Y. Peng W. Dong Z. Y. (2020). Long noncoding RNA H19 acts as a miR-340-3p sponge to promote epithelial-mesenchymal transition by regulating YWHAZ expression in paclitaxel-resistant breast cancer cells. Environ. Toxicol. 35 (9), 1015–1028. 10.1002/tox.22938 32420678

[B50] Yao N. Fu Y. Chen L. Liu Z. He J. Zhu Y. (2019). Long non-coding RNA NONHSAT101069 promotes epirubicin resistance, migration, and invasion of breast cancer cells through NONHSAT101069/miR-129-5p/Twist1 axis. Oncogene 38 (47), 7216–7233. 10.1038/s41388-019-0904-5 31444414

[B51] Ye J. Zhang R. Du X. Chai W. Zhou Q. (2019). Long noncoding RNA SNHG16 induces sorafenib resistance in hepatocellular carcinoma cells through sponging miR-140-5p. Onco. Targets. Ther. 12, 415–422. 10.2147/OTT.S175176 30655679 PMC6324603

[B52] Yeo S. K. Guan J. L. (2017). Breast cancer: Multiple subtypes within a tumor? Trends Cancer 3 (11), 753–760. 10.1016/j.trecan.2017.09.001 29120751 PMC5802368

[B53] Zeng L. Wang Q. Gu C. Yuan L. Xie X. He L. (2019). Asparagine synthetase and filamin A have different roles in ovarian cancer. Front. Oncol. 9, 1072. 10.3389/fonc.2019.01072 31681605 PMC6813569

[B54] Zhang K. Zhang L. Mi Y. Tang Y. Ren F. Liu B. (2020). A ceRNA network and a potential regulatory axis in gastric cancer with different degrees of immune cell infiltration. Cancer Sci. 111 (11), 4041–4050. 10.1111/cas.14634 32860283 PMC7648034

[B55] Zhao Y. Deng C. Lu W. Xiao J. Ma D. Guo M. (2011). let-7 microRNAs induce tamoxifen sensitivity by downregulation of estrogen receptor alpha signaling in breast cancer. Mol. Med. 17 (11-12), 1233–1241. 10.2119/molmed.2010.00225 21826373 PMC3321804

[B56] Zhou R. S. Zhang E. X. Sun Q. F. Ye Z. J. Liu J. W. Zhou D. H. (2019). Integrated analysis of lncRNA-miRNA-mRNA ceRNA network in squamous cell carcinoma of tongue. BMC Cancer 19 (1), 779. 10.1186/s12885-019-5983-8 31391008 PMC6686570

